# The Effect of Microbubble-Assisted Ultrasound on Molecular Permeability across Cell Barriers

**DOI:** 10.3390/pharmaceutics14030494

**Published:** 2022-02-24

**Authors:** Charis Rousou, Josanne de Maar, Boning Qiu, Kim van der Wurff-Jacobs, Marika Ruponen, Arto Urtti, Sabrina Oliveira, Chrit Moonen, Gert Storm, Enrico Mastrobattista, Roel Deckers

**Affiliations:** 1 Department of Pharmaceutical Sciences, Utrecht Institute for Pharmaceutical Sciences, Utrecht University, Heidelberglaan 8, 3584 CS Utrecht, The Netherlands; c.rousou@uu.nl (C.R.); b.qiu@uu.nl (B.Q.); k.m.g.vanderwurff-jacobs@uu.nl (K.v.d.W.-J.); s.oliveira@uu.nl (S.O.); g.storm@uu.nl (G.S.); 2 Imaging and Oncology Division, University Medical Center Utrecht, Heidelberglaan 100, 3584 CX Utrecht, The Netherlands; j.s.demaar-2@umcutrecht.nl (J.d.M.); c.moonen@umcutrecht.nl (C.M.); r.deckers-2@umcutrecht.nl (R.D.); 3 School of Pharmacy, Faculty of Health Sciences, University of Eastern Finland, Yliopistonranta 1 C, 70210 Kuopio, Finland; marika.ruponen@uef.fi (M.R.); arto.urtti@uef.fi (A.U.); 4 Division of Pharmaceutical Biosciences, Faculty of Pharmacy, University of Helsinki, Yliopistonkatu 4, 00100 Helsinki, Finland; 5 Institute of Chemistry, Saint Petersburg State University, Lieutenant Schmidt emb., 11/2, 199034 Saint Petersburg, Russia; 6 Cell Biology, Neurobiology and Biophysics, Department of Biology, Faculty of Science, Utrecht University, Heidelberglaan 8, 3584 CS Utrecht, The Netherlands; 7 Department of Surgery, Yong Loo Lin School of Medicine, National University of Singapore, 21 Lower Kent Ridge Rd, Singapore 119077, Singapore; 8 Department of Biomaterials Science and Technology, University of Twente, Drienerlolaan 5, 7522 NB Enschede, The Netherlands

**Keywords:** ultrasound, microbubbles, epithelial permeation, paracellular permeability, intracellular accumulation, nanobody

## Abstract

The combination of ultrasound and microbubbles (USMB) has been applied to enhance drug permeability across tissue barriers. Most studies focused on only one physicochemical aspect (i.e., molecular weight of the delivered molecule). Using an in vitro epithelial (MDCK II) cell barrier, we examined the effects of USMB on the permeability of five molecules varying in molecular weight (182 Da to 20 kDa) and hydrophilicity (LogD at pH 7.4 from 1.5 to highly hydrophilic). Treatment of cells with USMB at increasing ultrasound pressures did not have a significant effect on the permeability of small molecules (molecular weight 259 to 376 Da), despite their differences in hydrophilicity (LogD at pH 7.4 from −3.2 to 1.5). The largest molecules (molecular weight 4 and 20 kDa) showed the highest increase in the epithelial permeability (3-7-fold). Simultaneously, USMB enhanced intracellular accumulation of the same molecules. In the case of the clinically relevant anti- C-X-C Chemokine Receptor Type 4 (CXCR4) nanobody (molecular weight 15 kDa), USMB enhanced paracellular permeability by two-fold and increased binding to retinoblastoma cells by five-fold. Consequently, USMB is a potential tool to improve the efficacy and safety of the delivery of drugs to organs protected by tissue barriers, such as the eye and the brain.

## 1. Introduction

The functionality and vitality of tissues depend on a proper regulation of their barriers [1,2,3]. Tissue barriers are formed by layers of epithelial cells that separate organs from their environment, and endothelial cells in the vasculature (separating the bloodstream from the tissues). Examples of tissue barriers include the blood-retina barrier (BRB) in the posterior eye and the blood-brain barrier (BBB). The functions of these cellular barriers are critical for tissue, organ, and organism homeostasis. A factor of prime importance regulating their permeability is the presence of intercellular junctions between the cells [4]. Tight junctions and subjacent adherens junctions control barrier permeability and intercellular adhesive interactions. These barriers also hinder the delivery of drug molecules to the diseased tissues (e.g., in retinoblastoma in the eye, glioblastoma in the brain) and limit their therapeutic efficacy. New delivery methods that allow administered drugs to permeate across tissue barriers more efficiently, and in a controlled and safe manner, are therefore needed.

Ultrasound and microbubbles (USMB) have been previously investigated as a method to allow drug molecules to cross epithelial and endothelial barriers [5,6]. Microbubbles are gas-filled microspheres with diameters in the range of 0.5–10 μm. They are widely used as vascular contrast agents for diagnostic ultrasound imaging [7]. Microbubbles undergo mechanical oscillations when they are exposed to ultrasound waves. These oscillations are associated with various effects on cells and tissues. Some examples of these effects include (i) increase in the paracellular permeability as a consequence of rearrangement of the intercellular junctions; and (ii) enhancement of molecular permeation across cell membranes and intracellular accumulation as a result of pore formation (sonoporation) or enhanced endocytosis [8,9]. These effects have been exploited to increase the permeability of the BRB [10,11,12], the BBB [8,13] (including some clinical trials NCT03119961, NCT04417088, NCT04440358, NCT04528680), the blood-labyrinth barrier in the ear [14,15], and the skin barrier (epidermis) [16,17,18,19] for a variety of materials (small molecule drugs, antibodies, nanoparticles) that otherwise have limited permeation in these barriers. These studies focused on the importance of a single physicochemical feature of a drug, the molecular weight. However, the impact of other drug-related parameters (e.g., hydrophilicity) on USMB-enhanced barrier permeability is still unknown.

This study aimed to investigate the USMB-mediated transport efficacy of five different molecules with different (i) hydrophilicities and (ii) molecular weights across an epithelial cell barrier. Specifically, the chosen test molecules vary in octanol/water partition coefficient (LogD) at pH 7.4 between 1.5 (i.e., propranolol) and highly hydrophilic (i.e., dextrans), and in molecular weight between 182 Da and 20 kDa. The well-known experimental transwell model was used for studying the transport of the selected molecules across the in vitro barrier. Furthermore, the effect of USMB on the permeability of the barrier was investigated using a clinically relevant molecule as a model drug, namely an anti-C-X-C chemokine receptor type 4 (CXCR4) single domain antibody derived from a heavy chain only camelid antibody (also known as nanobody, molecular weight 15 kDa) [20,21]. To the best of our knowledge, this is the first time that the enhanced permeability of a nanobody across a biological barrier is studied using USMB. In clinical practice, USMB could be used to enhance the distribution of the anti-CXCR4 nanobody in the retina, and improve the efficacy of anti-cancer treatment in retinoblastoma.

## 2. Materials and Methods

### 2.1. Chemicals

Six-carboxyfluorescein (6-carboxyfluorescein, 8.51072), fluorescent-conjugated dextrans (4400 Da; T1037 and 20,000 Da; 73766) and histology mounting medium containing DAPI (Fluoroshield^TM^ with DAPI, F6057) were acquired from Sigma-Aldrich (Steinheim, Germany). Radioactive mannitol (^3^H-Mannitol, NET101250UC) and propranolol (^3^H-Propranolol, NET515250UC) were purchased from Perkin Elmer (Waltham, MA, USA). SYTOX^TM^ green (S7020) was obtained by ThermoFisher Scientific (Waltham, MA, USA). Fluorescently labelled anti-CXCR4 nanobody (anti-CXCR4-Hilyte647, molecular weight 15 kDa, Q85c-647) was obtained from QVQuality (Utrecht, The Netherlands).

### 2.2. Cell Culture

Madin-Darby canine kidney (MDCK) II is an epithelial cell line with short doubling time, and when cultured at specific conditions, cells acquire cobblestone morphology, cellular polarity, form microvilli and intercellular junctions (barrier function) [22,23]. MDCK II cells were kindly provided by Prof. Arto Urtti (University of Eastern Finland, Kuopio, Finland) and were maintained in DMEM/F12 medium (Gibco, New York, NY, USA) supplemented with 10% (*v*/*v*) FBS and 1% (*v*/*v*) L-glutamine (Sigma-Aldrich, Steinheim, Germany). Human retinoblastoma cells (WERI-RB1) were purchased from ATCC (ATCC, Wesel, Germany) and were cultured in RPMI 1640 medium (Sigma-Andrich) supplemented with 10% (*v*/*v*) FBS.

Cells were cultured in standard cell culture flasks in a humidified atmosphere of 5% CO_2_ at 37 °C. Epithelial cells were sub-cultured two to three times per week in dilution ratios between 1:5–1:20 and were used up to passage number 32. Sub-culture of epithelial cells involves washing the cells with DPBS, incubation with trypsin/EDTA for 5 min at 37 °C and deactivation of trypsin/EDTA with normal culture medium containing all of the supplements. Retinoblastoma cells were sub-cultured one to two times per week and were used up to passage number seven. Sub-culture of retinoblastoma cells (suspension cells) was performed by diluting cells into fresh medium with cell density maintained between 0.1 and 2 × 10^6^ cells/mL.

### 2.3. Differentiation of Epithelial Cells

**Figure 1:**
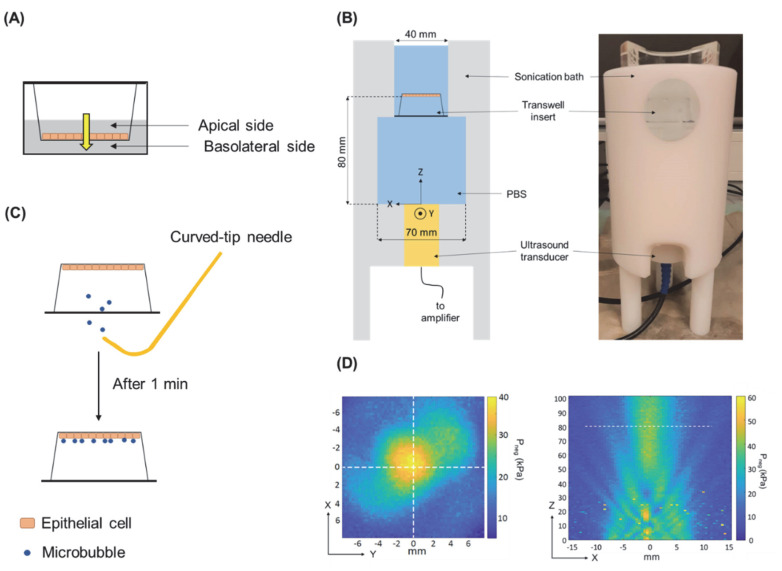
(**A**) Apical and basolateral sides of transwell insert and direction in which the permeability of model drugs was studied (yellow arrow). (**B**) Schematic illustration (left) and picture (right) of the custom-made sonication bath, compatible with transwell inserts. The ultrasound transducer is positioned at the bottom of the bath and the transwell insert is immersed upside-down with the cell monolayer fixed at 80 mm from the surface of the transducer. (**C**) Using a needle with curved tip, microbubbles are injected and allowed to float for 1 min to ensure cell-microbubble contact. (**D**) Pressure field maps of ultrasound transducer. Left: transversal plane. Right: axial plane, white dotted line indicates position of transversal plane. Adapted from [26], Frontiers Media S.A., 2021.

For the permeability studies, epithelial cells were cultured as tight monolayers on transwell membranes. Cells were seeded to polycarbonate transwell membranes (12 or 24 mm translucent membrane, pore size 0.4 µm, Corning, New York, NY, USA) at the density of 0.16 × 10^6^ cells/cm^2^. Prior to cell seeding, the transwell membranes were equilibrated for 10 min with culture medium at room temperature. To maintain equilibrium of hydrostatic pressure between the apical and the basolateral sides of the transwell membrane (Figure 1A), insert medium was added to the recommended final volumes according to the manufacturer (apical/basolateral: 0.5/1.5 mL for 12 mm membranes or 1.5/2.6 mL for 24 mm membranes). Medium containing 1% FBS and 1% antibiotics was used during cell seeding, which was refreshed one, three, and five days after seeding. Cells were cultured for 7 days prior to the experiment to allow for differentiation and development of intercellular junctions, as previously described [22].

### 2.4. USMB Treatment of Epithelial Barriers

The permeability of various test compounds across epithelial monolayers cultured on transwell membranes was studied in the apical-to-basolateral direction (Figure 1A). On the experimental day, fully differentiated epithelial monolayers were washed twice and equilibrated for 10 min at 37 °C in Hank’s Balanced Salt Solution (HBSS) containing calcium and magnesium without phenol red (ThermoFisher Scientific, Waltham, MA, USA).

Ultrasound experiments were performed using an unfocused, single-element transducer with 20 mm diameter (PA420, Precision Acoustics, Dorchester, UK). The arbitrary wave generator of an oscilloscope (SDS1202X+, Siglent.eu, Helmond, The Netherlands) was used to generate a transistor-transistor logic (TTL) pulse, which was used as an external trigger for an arbitrary wave generator (SDG1032X 30Mhz, Siglent.eu), resulting in an output pulsed sinusoidal signal with central frequency 1.5 MHz, pulse duration 100 μs, duty cycle 10% and pulse repetition frequency (PRF) 1 kHz. The chosen transducer has a central frequency that falls within the range of resonance frequencies of SonoVue^TM^ [24], which ensures that microbubble oscillations are maximum during USMB treatment. The resulting signal was then amplified (AG1012, T&C Power Conversion Inc., New York, NY, USA) before it was sent to the ultrasound transducer.

A dedicated sonication tank compatible with transwell inserts was used for the USMB experiments (Figure 1B). The ultrasound transducer was positioned at the bottom of the bath and about 250 mL of PBS at 37 °C was added. Subsequently, the transwell insert was immersed in the bath with the transwell membrane covered with cells facing down (Figure 1B) at a fixed axial distance (80 mm) from the ultrasound transducer.

Transwell inserts were randomly assigned to USMB-treated or sham-treated sample groups. Following immersion of USMB-treated samples in the sonication bath, 500 μL of freshly mixed SonoVue^TM^ microbubbles (Bracco, Milan, Italy) was injected near the cells using a curved-tip 19G needle (Figure 1C). SonoVue^TM^ are lipid-shelled microbubbles with mean diameter of 2.5 μm, that contain sulfur hexafluoride gas (SF_6_) and are approved for clinical use [25]. Microbubbles were allowed to float for 1 min so that direct contact with the cells was assured. Subsequently, ultrasound was applied for 1 min with acoustic peak negative pressure (P_neg_) ranging between 0.3 and 0.7 MPa. Sham-treated samples were treated similar to USMB-treated samples, except for the addition of microbubbles and exposure to ultrasound. Sham-treated samples were immersed in the PBS bath for 2 min and served as negative controls (0 MPa). The pressure field maps (Figure 1D) of the transducer were measured with a calibrated hydrophone [26].

### 2.5. Permeability Experiments with an Epithelial Barrier and Model Drugs

Immediately after USMB treatment, transwell inserts were removed from the sonication bath (containing PBS) and were placed in a new six-well plate. The protocol used in the permeability experiments was according to similar studies previously performed by others [22,27]. In short, HBSS was added at the basolateral side followed by addition of test compound dissolved in HBSS (1.5 mL) at the apical side. The initial concentration of test compounds on the apical side was 1 μCi/mL for propranolol and mannitol and 200 μM for 6-carboxyfluorescein and fluorescent dextrans. An overview of the relevant physicochemical characteristics of the test compounds used in the permeability experiments is given in Table 1.

To determine compound concentration on the basolateral side, samples of 400 μL were collected at 15, 30, 45, 60, 90 and 120 min after treatment with USMB. The volume of removed sample was replaced with fresh HBSS. During the experiment, cells were kept at 37 °C and shaking at 170 rounds per minute. In the experiments where fluorescent compounds were used, samples were protected from light at all times.

The samples containing radioactively-labelled compounds were equilibrated overnight in liquid scintillation cocktail for radiometric detection (Ultima Gold, 6013321, Perkin Elmer) at room temperature. Detection of radioactivity counts was determined by a microplate counter for radiometric counting (MicroBeta, 2450, Perkin Elmer).

Intensity of fluorescence signals was determined by a spectrofluorophotometer (FP 8300, Jasco Benelux BV, Utrecht, The Netherlands). 6-carboxyfluorescein was excited at 492 nm and emission was detected at 517/5 nm (center wavelength/bandwidth) and TRITC-dextrans were excited at 550 nm and detected at 575/5 nm. Background radioactivity and fluorescence signal from HBSS were subtracted from the measurements.

Calculation of apparent permeability coefficients (P_app_):

P_app_ values were calculated for each compound:

(1) $$P_{\mathrm{app}}=\frac{Q}{\left(C_{0}\times A\right)},$$

where P_app_ (in cm/s) is the apparent permeability coefficient of the test compound across the barrier. Q is the flux of the test compound (µg/s), C_0_ is the initial concentration of the test compound at the apical side (µg/mL), and A is the surface area of the transwell membrane (cm^2^). In addition to P_app_, the amount of permeated compound across the cell barrier was calculated as a percentage of the amount of the compound initially added at the apical side (Equation (2)).

(2) $$\%\;\mathrm{Permeated}\;\mathrm{amount}=\frac{{\mathrm{Amount}}_{B\left(t\right)}}{{\mathrm{Amount}}_{A\left(t=0\right)}}\times 100,$$

where Amount_B(t)_ is the amount of the compound at the basolateral side at time t and Amount_A(t=0)_ is the amount of the compound added at the apical side at the beginning of the experiment (t = 0).

### 2.6. Intracellular Accumulation Study

Epithelial cells were exposed to acoustic pressures of 0 MPa or 0.7 MPa. Directly after USMB treatment, a solution containing 200 μM of 4 kDa or 20 kDa fluorescent dextran and 2 μΜ of SYTOX^TM^ green was added at the apical side. SYTOX^TM^ green is a small (molecular weight 600 Da) membrane impermeable compound, commonly used as a drug model in studies investigating the effect of USMB on intracellular drug accumulation. Here, we use SYTOX^TM^ green to confirm the action of USMB when investigating the intracellular accumulation of dextrans. As with SYTOX^TM^ green, dextrans are hydrophilic in nature, thus unable to permeate the lipophilic cell membrane as such. When USMB induces cell membrane disruption, these molecules can enter the cytoplasm.

After 30 or 120 min of incubation with the test compounds at 37 °C, the transwell membrane was removed from the insert with a scalpel blade. The cells were washed with PBS and fixed with 4% (*w*/*v*) PFA (Sigma-Aldrich) in PBS for 10 min at room temperature. Subsequently, the transwell membrane was positioned on a glass microscope slide with the cells facing up. A small amount of mounting medium containing DAPI was added on a cover slip, which was used to seal the transwell membrane and the glass microscopy slide. Samples were allowed to dry overnight and were kept at 4 °C until further use.

Fixed cells were imaged using a fluorescence confocal microscope (Leica TCS SP8 X, Leica, Amsterdam, The Netherlands) in three channels (excitation 360 nm; emission 410–480 nm for DAPI, excitation 504 nm; emission 515–546 nm for SYTOX^TM^ green, excitation 550 nm; emission 565–650 nm for TRITC-dextrans). Samples were initially imaged in the DAPI and SYTOX^TM^ green channels at 10× magnification (image format: 2048 × 2048 pixels; speed: 100; line average: 4) in order to confirm that USMB acted on cell membranes and to detect the locations where this activity was the most dense (Supplementary Figure S1A). Subsequently, cells at these locations were imaged with a 63× oil immersion objective (image format: 2048 × 2048 pixels, speed: 100, line average: 6) to determine the intracellular accumulation of TRITC-dextrans (Supplementary Figure S1B). All imaging settings (laser gain, pinhole size, zoom) remained constant among different samples.

The fluorescence intensity (FI) of cells that showed intracellular accumulation of dextrans was determined with ImageJ software (National Institutes of Health, Bethesda, MD, USA). A mask was generated by thresholding the original RGB TRITC-dextran image at a fixed pixel intensity. Threshold value was kept the same for all images for each of the two dextrans. Subsequently, holes in the mask were filled automatically using the corresponding software command. The mask was applied to the RGB image to calculate the mean FI of cells within the area of interest. An example of the resulted image for each of the above steps can be found in the supplementary information (Supplementary Figure S2). For each experimental condition images from five different locations were acquired and the average FI intensity per cell with intracellular accumulation was calculated for each experimental group.

### 2.7. USMB-Induced Permeability of Anti-CXCR4 Nanobody across an Epithelial Barrier

Immediately after exposure of epithelial barrier to USMB (Section 2.5), 0.5 mL of HBSS containing anti-CXCR4 nanobody (1000 nM) was added to the apical side of the transwell. Hydrostatic equilibrium was maintained with the addition of 1.5 mL of RPMI 1640 medium without phenol red (Gibco, New York, NY, USA) in the basolateral side. The cells were incubated for 120 min in a humidified atmosphere of 5% CO_2_ at 37 °C, and when incubation was complete, medium from the basolateral side containing the permeated nanobody was collected.

To determine the amount of permeated fluorescent nanobody, 0.5 mL of basolateral medium was measured using a fluorescence plate-reader (FP 8300, Jasco, excitation 650 nm, emission 673/5 nm). Subsequently, 1 mL of the remaining basolateral medium was added to retinoblastoma cells (100,000 cells in Eppendorf tube) allowing for binding of nanobody on CXCR4 receptor for 1 h at 5% CO_2_ and 37 °C. Then, the cells were centrifuged (1000× *g*, 4 min) and supernatant was removed. Cell pellets were washed once with PBS and fixed with 4% PFA for 10 min at room temperature. After fixation and removal of PFA, the cell pellets were suspended in PBS, stored at 4 °C and were protected from light until further analysis. Nanobody binding was determined by measuring the fluorescence of cells using the same plate-reader and settings as before, and flow cytometry (excitation 633 nm, emission 780/60 nm, FACSCanto™ II Cell Analyzer, BD Biosciences, Franklin Lakes, NJ, USA). Flow cytometry data were analyzed using FlowLogic^TM^ software (V8, Inivai, Mentone, Victoria, Australia).

### 2.8. Statistical Analysis

Statistical analysis was performed using the GraphPad Prism software (version 8.0.1, GraphPad, San Diego, CA, USA), assuming that the samples follow non-parametric distribution. Statistically significant differences in P_app_ and percentage of permeated amount were calculated between USMB-treated and sham-treated samples (i.e., 0.3–0.7 MPa vs. 0 MPa) using Kruskal-Wallis test. Differences in the normalized IF of intracellular TRITC-dextran accumulation of 0 MPa vs. 0.7 MPa, and permeability and binding of the anti-CXCR4 nanobody were calculated using Mann-Whitney test. Data in the graphs are shown as mean ± SEM. Statistically significant differences between groups are annotated with asterisks by using * for *p* < 0.05; ** for *p* < 0.01; *** for *p* < 0.001.

## 3. Results

### 3.1. Effect of USMB on Molecular Permeability across an Epithelial Barrier

**Figure 2:**
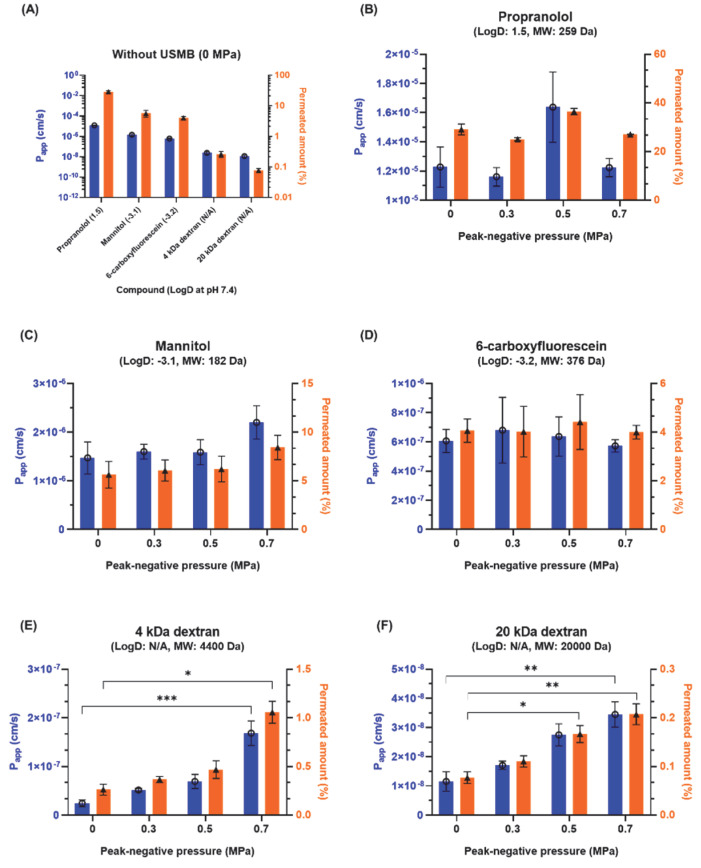
Apparent permeability coefficient (blue bars, **◯**) and total amount permeated (orange bars, ▲) of five different molecules with varying molecular weight and hydrophilicity across an epithelial barrier (**A**) in the absence of USMB (0 MPa) and (**B**–**F**) at various ultrasound pressures for (**B**) propranolol, (**C**) mannitol, (**D**) 6-carboxyfluorescein, (**E**) 4 kDa dextran, and (**F**) 20 kDa dextran (*n* = 5), * for *p* < 0.05; ** for *p* < 0.01; *** for *p* < 0.001.

Permeability experiments were conducted using molecules with varying molecular weight and hydrophilicity. In the absence of USMB permeability was highest for propranolol and decreased with increasing molecular weight and decreasing LogD (Figure 2A). More specifically, the total permeated amount was 29.1 ± 2.2% for lipophilic propranolol, followed by hydrophilic mannitol (5.6 ± 1.4%), 6-carboxyfluorescein (4.1 ± 0.5%), 4 kDa dextran (0.3 ± 0.1%), and 20 kDa dextran (0.1 ± <0.1%).

Treatment of cells with USMB at increasing ultrasound pressures did not have a significant effect on the permeability of small molecules (propranolol, mannitol, 6-carboxyfluorescein), despite their differences in hydrophilicity (Figure 2B–D). In contrast, for the large hydrophilic molecules (dextrans), an increase in permeability was observed with increasing ultrasound pressure (Figure 2E,F). For the 4 kDa dextran, a significant, seven-fold increase in the mean P_app_ was seen at 0.7 MPa (1.68 × 10^−7^ ± 0.25 × 10^−7^ cm/s) compared to 0 MPa (0.24 × 10^−7^ ± 0.07 × 10^−7^ cm/s) (Figure 2E). At the same pressure, the total amount of 4 kDa dextran that permeated the barrier 120 min after treatment was four times higher than the sham treatment (0.3 ± 0.1% at 0 MPa vs. 1.1 ± 0.1% at 0.7 MPa). Similarly, a three-fold increase in P_app_ was observed for the 20 kDa dextran (1.15 × 10^−8^ ± 0.33 × 10^−8^ cm/s at 0 MPa vs. 3.44 × 10^−8^ ± 0.43 × 10^−8^ cm/s at 0.7 MPa) (Figure 2F). The total amount of 20 kDa dextran that permeated the barrier was increased by two and three times at 0.5 MPa and 0.7 MPa, respectively, compared to 0 MPa (0.1 ± <0.1% at 0 MPa, 0.2 ± <0.1% at 0.5 MPa, 0.2 ± <0.1% at 0.7 MPa). The results indicate that USMB aided the permeability of the two large hydrophilic molecules, but this effect was absent in the case of small molecules, regardless of their hydrophilicity.

In addition to the permeability experiments using epithelial barriers, similar experiments were performed with an endothelial cell line (HUVEC) (Supplementary Information Sections 1.1–1.5 and 2.1). USMB treatment of endothelial barriers at ultrasound pressures of 0.6 and 0.7 MPa led to severe cell detachment and disruption of barrier integrity (Supplementary Figure S6A). Therefore, permeability experiments were only performed at lower ultrasound pressures (0.3 to 0.5 MPa) (Supplementary Figure S6B). Comparison of the permeability coefficients between the MDCK II and HUVEC cells revealed that HUVEC formed a leakier barrier than MDCK II. Specifically, in the absence of USMB, the P_app_ of 6-carboxyfluorescein, 4 kDa, and 20 kDa dextrans was several times higher (~20 times) than the corresponding values from the MDCK II cells.

### 3.2. The Effect of USMB on the Intracellular Accumulation of Fluorescent Dextrans in Epithelial Cells

To investigate whether USMB-induced intracellular accumulation occurred in parallel with USMB-enhanced paracellular transport, epithelial barriers were treated with USMB at 0.7 MPa, the ultrasound pressure that was found to increase the paracellular permeability of large hydrophilic molecules (dextrans) across the same barrier (Section 3.1). Intracellular accumulation was investigated by incubating cells with the same two fluorescent dextrans (TRITC-dextrans, molecular weight of 4 or 20 kDa). Furthermore, SYTOX^TM^ green was added to confirm that intracellular accumulation of dextrans was induced by USMB. It was observed that all cells with uptake of dextrans had concomitant uptake of SYTOX^TM^ green (Supplementary Figure S1B).

**Figure 3:**
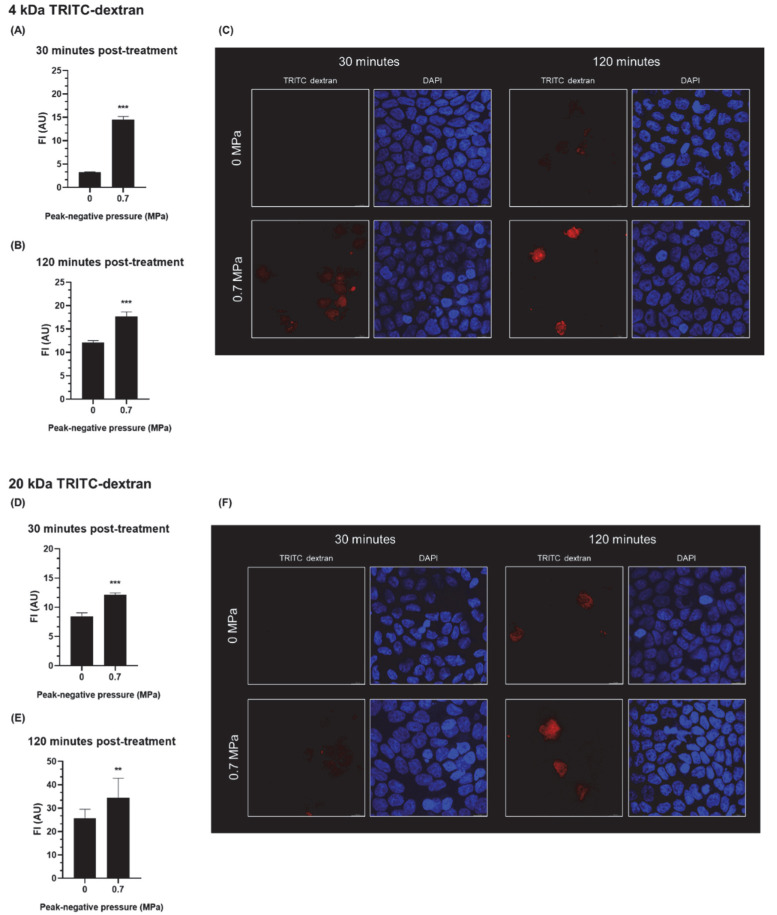
Intracellular accumulation of fluorescent dextrans (4 and 20 kDa) by epithelial barriers after treatment with USMB at acoustic pressure of 0 or 0.7 MPa. Quantification of fluorescence intensity from the intracellular accumulation of a 4 kDa dextran after incubation for (**A**) 30 min and (**B**) 120 min. (**C**) Representative fluorescence images used for the quantification, scale bar, 10 μm. Quantification of fluorescence intensity from the intracellular accumulation of a 20 kDa dextran after incubation for (**D**) 30 min and (**E**) 120 min. (**F**) Representative fluorescence images used for the quantification, scale bar, 10 μm. FI, fluorescence intensity; AU, arbitrary units (*n* = 5); ** for *p* < 0.01; *** for *p* < 0.001.

Quantification of fluorescent signal revealed that for both dextrans the intracellular accumulation was lowest in the absence of USMB after 30 min of incubation (Figure 3A,D). Exposure of cells to USMB significantly increased the uptake of dextrans at both time points. Specifically, 30 min after incubation the intracellular accumulation of 4 kDa dextran (4.4 times, Figure 3A) and 20 kDa dextran (1.4 times, Figure 3D) was increased. Two hours after incubation, sham treated cells had intracellular accumulation of dextrans to some extent (Figure 3C,F), yet USMB-induced uptake was significantly higher (1.5 times increase in the uptake of a 4 kDa dextran, 1.3 times increase in the uptake of a 20 kDa dextran).

### 3.3. Effect of USMB on the Permeability of Anti-CXCR4 Nanobody across an Epithelial Barrier

As previously demonstrated (Section 3.1), USMB aided paracellular permeability of the hydrophilic dextrans at ultrasound pressure of 0.7 MPa. To demonstrate the clinical relevance of USMB treatment, experiments were performed using a model therapeutic molecule. A nanobody that binds on the CXCR4 was chosen for this purpose [20,21]. CXCR4 is overexpressed in a number of cancer cells including retinoblastoma cells in the eye [30]. Since epithelial cells used in this study did not express CXCR4 receptors (Supplementary Figure S3), any increase in the permeability of the anti-CXCR4 nanobody across the epithelial monolayer is a result of USMB treatment, but not of receptor-mediated transcytosis.

**Figure 4:**
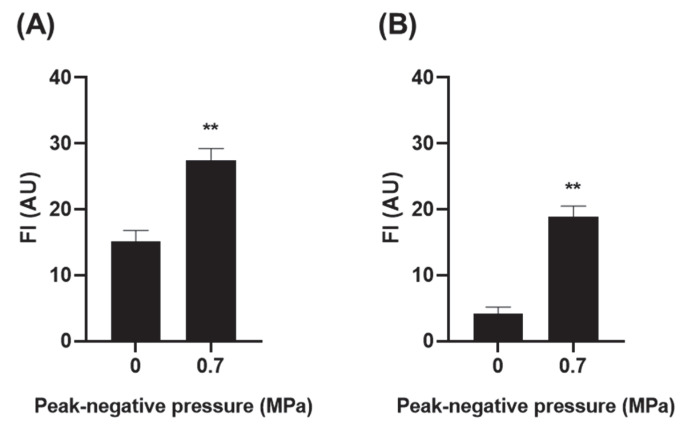
(**A**) Permeability of anti-CXCR4 nanobody across the epithelial barrier upon USMB treatment at acoustic pressure of 0 or 0.7 MPa and (**B**) binding of permeated nanobody to retinoblastoma cells. FI: fluorescence intensity, AU: arbitrary units (*n* = 5), ** for *p* < 0.01.

Epithelial monolayers were treated with USMB at 0 MPa or 0.7 MPa, and subsequently the anti-CXCR4 nanobody (fluorescently labelled) was added at the apical side. Exposure of epithelial barrier to USMB increased the amount of permeated nanobody by 1.8 times (FI 15.12 ± 1.65 AU at 0 MPa vs. 27.48 ± 1.72 AU at 0.7 MPa) (Figure 4A). To check that the permeated nanobody retained its ability to bind on the CXCR4 receptor after permeating the barrier, the solution from the basolateral side containing the permeated nanobody was collected and incubated with the retinoblastoma cells. Exposure of epithelial cells to USMB at 0.7 MPa allowed for an increase in the total nanobody binding to retinoblastoma cells of 4.5 times (FI 4.17 ± 0.99 AU at 0 MPa vs. 18.81 ± 1.67 AU at 0.7 MPa) (Figure 4B). This increase in the binding of nanobody was also observed by flow cytometry (Supplementary Figure S4).

## 4. Discussion

The objective of this study was to investigate how USMB affects the permeability of molecules with varying physicochemical properties (hydrophilicity and molecular weight) in biological barriers. Permeability of the lipophilic propranolol (molecular weight 259 Da, LogD at pH 7.4 of 1.5) was the highest among the studied molecules and was not changed due to USMB treatment. This is attributed to the lipophilicity of propranolol that allows its high permeation across cellular barriers via transcellular diffusion.

In vitro cell cultures are used as models of in vivo biological barriers, which often means that they resemble (but do not perfectly preserve) the characteristics of their in vivo counterparts. All in vitro epithelial models exhibit some leakiness even for hydrophilic molecules that do not cross the membranes via transcellular diffusion [31]. Small hydrophilic molecules can permeate through the intercellular spaces easier than large molecules. Indeed, in the absence of USMB, P_app_ of mannitol (molecular weight 182 Da, LogD at pH 7.4 of −3.1) in MDCK II monolayer was one order of magnitude higher than the permeability of 6-carboxyfluorescein (molecular weight 376 Da, LogD at pH 7.4 of −3.2) and 4 kDa dextran, and two orders of magnitude higher than the permeability of 20 kDa dextran. The lack of measurable effect on the paracellular permeability of mannitol (molecular radius ≈ 4 Å [32]) and 6-carboxyfluorescein is due to this intrinsic permeability that likely overshadowed cell barrier alterations as induced by USMB. On the contrary, clearly improved permeation of the dextrans (several fold) was achieved with USMB. Presumably, the small size of paracellular pores in the differentiated MDCK II monolayers (radii of pores 5–10 Å; [33]) was increased by USMB thereby facilitating paracellular diffusion of the two dextrans (molecular radius of 4 kDa dextran ≈ 11 Å and of 20 kDa dextran ≈ 18 Å [34]). It is important to highlight that USMB did not induce any reduction in the viability of epithelial cells or the integrity of the barrier after exposure at this ultrasound pressure (Supplementary Figure S5).

Only few other studies have investigated the effect of USMB on the transport of different molecules through intercellular gaps using the transwell system. In agreement with our findings, Fix et al. reported the effect of USMB in the paracellular permeability of a hydrophilic macromolecule (70 kDa dextran) across an epithelial CaCo-2 barrier on transwells [35]. Exposure to ultrasound (frequency 1 MHz, P_neg_ 0.3 MPa, 30 s) combined with phase-change contrast agents (i.e., liquid perfluorocarbon-filled particles of size 100–300 nm that form microbubbles) increased the paracellular permeability of a 70 kDa dextran by 44%. Lelu et al. cultured primary porcine brain endothelial cells (PBEC) on transwell inserts as an in vitro model for BBB [36]. The barriers were exposed to USMB at 0.1 and 0.8 MPa and permeability of two fluorescent molecules (molecular weight of 0.46 and 67 kDa) was measured across the endothelial barrier. The authors reported a significant increase of P_app_ for both molecules (2.6–5.2 times). Lelu et al. [36] reported that immersion of the cells in PBS for USMB treatment affected the tightness of the barrier, which is in agreement with our observations (data not shown). Based on this observation we decided to perform all statistical comparisons in our study with sham treated sample (i.e., cells that were immersed in PBS bath but not exposed to USMB) as a control group rather than completely untreated barriers. This difference between the control group used in our study (sham-treated barriers) versus the study of Lelu et al. [36] (untreated barriers), unfortunately makes comparison and interpretation of results difficult.

In addition to increasing the intercellular permeability of hydrophilic molecules across the epithelial barrier, USMB induced intracellular accumulation of SYTOX^TM^ green (molecular weight 600 Da) and two dextrans (molecular weight 4 and 20 kDa) as was already shown by us [26,37] and other groups [38,39,40] before. Quantification of fluorescent signals 30 and 120 min after incubation revealed that intracellular accumulation of dextrans was significantly higher in the cells treated with USMB at 0.7 MPa as compared to 0 MPa. Dextrans were distributed homogeneously in the cytosol of USMB-treated cells, presumably due to cellular access via USMB-induced pores in plasma membrane [40]. Nuclear distribution of 4 kDa dextran was observed as early as 30 min after incubation, while the presence of the 20 kDa dextran in the nucleus was only observed after 120 min. Meijering et al. observed similar distribution of a 4 kDa dextran in the cell nucleus (immediately after treatment with USMB), but not with dextrans larger than 70 kDa [40], which is due to the effective porous radius of the nuclear membrane (macromolecules larger than ~70 kDa do not cross the nuclear pore complex by passive diffusion but rather require energy-dependent processes [41]).

A few studies have reported accumulation of full-length antibodies to the brain aided by USMB [13,42,43]. Nanobodies, which are single-domain antibodies, have been previously coupled to microbubbles and were used to improve ultrasound molecular imaging of atherosclerosis, prostate and renal tumors [44,45,46]. Here, a nanobody (molecular weight 15 kDa) targeting the CXCR4 receptor [20,21] was used as a model molecule to illustrate the potential of USMB in the delivery of pharmaceuticals across epithelial barriers. CXCR4 is a receptor that is highly expressed in over than 20 cancer types and is a major co-receptor for cellular entry of human immunodeficiency virus [47]. It is involved in tumor cell proliferation, survival and metastasis, while it is absent or expressed at very low levels in healthy tissues [48,49]. These characteristics make CXCR4 inhibitors an interesting compound group for the treatment of those malignancies. Recently, results from a phase Ib/II trial using a fully human IgG4 monoclonal anti-CXCR4 antibody (Ulocuplumab, BMS-936564) were published, showing safe and efficacious clinical use of Ulocuplumab against multiple myeloma [50]. Expression of CXCR4 was previously observed in tumor cells isolated from retinoblastoma [30], the most common intraocular malignancy in children. In our study, treatment of epithelial barriers at ultrasound pressure of 0.7 MPa increased the total number of permeated nanobody approximately by 2 times compared with sham-treated barriers, and resulted in 4.5 times higher binding of nanobody to retinoblastoma cells. In clinical practice, USMB could be used to improve the permeability of anti-CXCR4 nanobody across tissue barriers that limit its binding to the target cells. For example, USMB might make it possible to deliver anti-CXCR4 nanobody to retinoblastoma cells, allowing for increased nanobody binding to tumor cells and inhibiting their migration and proliferation. The nanobody could be administered intravenously and USMB is used to temporarily increase the permeability of the BRB or to facilitate nanobody permeation to retinoblastoma cells after intravitreal injection of the nanobody.

In addition to the permeability experiments with the epithelial cells (MDCK II), we performed similar experiments using an endothelial cell line (HUVEC). Comparison of the permeability coefficients between the MDCK II and HUVEC cells revealed that HUVEC formed a leakier barrier than MDCK II. This inherent leakiness of HUVEC explains why no USMB-enhanced permeability was detected when endothelial barriers were treated with USMB. In addition, exposure of HUVEC barriers to acoustic pressures higher than 0.6 MPa resulted in extensive cell detachment from the transwell membrane, making permeability experiments at these pressures practically impossible. Detachment of HUVEC after exposure to USMB was previously reported at similar ultrasound pressures [37]. In conclusion, cell detachment in combination with insufficient barrier tightness [51] make HUVEC not an ideal endothelial barrier model for investigating the effect of USMB on molecular permeability. In future studies, co-culture of HUVEC with other supportive cells, such as pericytes, could improve the tightness of the barrier and provide a more suitable model for permeability studies [52,53].

Our study has some limitations. Our sonication bath set-up did not allow for the integration of cavitation measurements. Real-time monitoring of acoustic emissions from microbubbles could provide additional insights on which mechanisms (i.e., stable vs. inertial cavitation) are responsible for para- and intracellular transport. In this study we only investigated the effect of acoustic pressure on barrier permeability but future studies should focus on the effect of other USMB-related parameters (e.g., microbubble concentration, exposure time, PRF, etc.). Previous in vivo studies have shown that increase in the paracellular diffusion of compounds after USMB treatment is on the time scale of several hours [54,55]. This is an interesting aspect to be investigated in future studies using epithelial barriers and the transwell system.

The effect of USMB on the permeability of the BRB has not been yet extensively investigated. Experiments using ex vivo eyes or healthy animals could provide further insights on the safety of USMB prior to clinical translation for drug delivery in the posterior eye. A few in vivo studies that investigated the extravasation of molecules with different molecular weights as a result of USMB-mediated BRB disruption, are currently available [10,11,12]. New studies using molecules with various hydrophilicities and molecular weights could help to comprehend the potential and limitations of USMB therapy in ocular drug delivery. Ideally, clinically approved microbubbles and a clinical ultrasound system could be used for USMB treatment [26] and to monitor microbubble emissions [56]. Finally, combination of USMB with pharmaceuticals (such as an anti-CXCR4 nanobody for the treatment of retinoblastoma) could be tested in in vivo disease models in order to determine the therapeutic efficacy of the method.

## 5. Conclusions

The role of molecular properties (molecular weight and hydrophilicity) on the intercellular permeation across an epithelial barrier was studied in the presence of USMB. USMB at 0.7 MPa aided the paracellular permeability of large hydrophilic molecules and increased their intracellular accumulation, but did not affect the permeability of small molecules regardless of their hydrophilicity. USMB enhanced the paracellular permeability of an anti-CXCR4 nanobody and its subsequent binding to retinoblastoma cells. USMB is a potential tool for the delivery of (biological) drugs to protected organs, such as the eye and the brain.

## Figures and Tables

**Table 1:** Physicochemical properties of compounds used to study the permeability across epithelial barriers.

Compound	Hydrophilic/Lipophilic (LogD)	M_W_ (Da)	Label
Propranolol	Lipophilic (1.5 [28])	259	Radioactive
Mannitol	Hydrophilic (−3.1 [28])	182	Radioactive
6-carboxyfluorescein	Hydrophilic (−3.2 [29])	376	Fluorescent
4 kDa dextran	Hydrophilic (N/A)	4400	Fluorescent (TRITC)
20 kDa dextran	Hydrophilic (N/A)	20,000	Fluorescent (TRITC)

LogD, octanol/water partition coefficient at pH 7.4; N/A, not available; M_W_, molecular weight; TRITC, tetramethylrhodamine isothiocyanate.

## Data Availability

All data available are reported in the article.

## Supplementary Materials

The following are available online at https://www.mdpi.com/article/10.3390/pharmaceutics14030494/s1, Figure S1: (A) Fluorescence image of epithelial (MDCK II) cells showing an area with intracellular accumulation of SYTOX^TM^ green after treatment with USMB at 0.7 MPa. Cell nuclei stained with DAPI. Scale bar: 100 μm (B) SYTOX^TM^ green positive cells (left) with simultaneous uptake of TRITC-dextran (middle) and overlay with DAPI (right). Scale bar: 10 μm, Figure S2: Summary of steps followed for the quantification of intracellular accumulation of TRITC-dextrans in epithelial (MDCK II) cells. (A) Raw RGB image used to calculate fluorescence intensity in the regions of interest, (B) Thresholding of RGB image, (C) generation of mask with regions of interest (black areas). Scale bar: 10 μm, Figure S3: (A) Binding of anti-CXCR4 nanobody to retinoblastoma (WER-RB1) and epithelial (MDCK II) cells for nanobody concentrations of 0–1500 nM. (B) Magnification of area shown in red box in (A). FI: fluorescence intensity, Figure S4: Binding of permeated anti-CXCR4 nanobody in retinoblastoma cells measured with flow cytometry. Nanobody was collected from the basolateral side of epithelial barriers treated with USMB at 0 or 0.7 MPa. ns: not significant (*n* = 5), Figure S5: (A) Percentage of viable epithelial cells exposed to USMB at different ultrasound pressures (0.3–0.7 MPa) as compared to sham-treated cells (*n* = 3). Cell viability was studied using the alamarBlue^TM^ (ThermoFisher Scientific) assay after incubation of cells with resazurin solution for 120 min. (B) Fluorescence microscopy images of epithelial cell nuclear staining (DAPI) acquired at the edge (left column) and center (right column) of transwell membranes. No alterations in the integrity of the epithelial barrier were seen at 0.5 or 0.7 MPa. Uniform cell distribution without cell detachment was observed, Figure S6: (A) Brightfield microscopy images of endothelial (HUVEC) barriers acquired before (left) after (right) exposure to USMB. Treatment of cells at acoustic pressures of 0.6 and 0.7 MPa resulted in extensive cell detachment and disruption of monolayer integrity. (B) Apparent permeability coefficient (blue bars, ◯) and total amount permeated 120 min post-treatment (orange bars, ▲) of 6-carboxyfluorescein, 4 kDa dextran and 20 kDa dextran after treatment of endothelial cell monolayer with USMB at various acoustic pressures (*n* = 3).
