# Supplementary material for: The Effect of Microbubble-Assisted Ultrasound on Molecular Permeability across Cell Barriers
Source: Pharmaceutics. 2022 Feb 24;14(3):494. doi: 10.3390/pharmaceutics14030494 (PMC8949944; doi:10.3390/pharmaceutics14030494)
Supplement: Supplementary file 1 [file pharmaceutics-14-00494-s001.zip › pharmaceutics-1589877-supplementary.pdf]

pharmaceutics

Supplementary Information: The Effect of Microbubble-Assisted Ultrasound on Molecular Permeability across Cell Barriers

Charis Rousou, Josanne de Maar, Boning Qiu, Kim van der Wurff-Jacobs, Marika Ruponen, Arto Urtti, Sabrina Oliveira, Chrit Moonen, Gert Storm, Enrico Mastrobattista and Roel Deckers

SI-1. Materials and methods

SI-1.1. Culture of endothelial cells

Human umbilical vein endothelial cells (HUVEC) were acquired from Lonza (Verviers, Belgium) and cultured in Endothelial Basal Medium (EBM-2) supplemented with growth factors (Growth Medium 2 SupplementMix®, PromoCell, Heidelberg Germany) and 0.1% antibiotics (gentamicin/amphotericin B, Gibco, New York, USA). Cells were sub-cultured 1-2 times per week in dilution ratios between 1:3-1:6 and were used up to passage number 7.

SI-1.2. Differentiation of endothelial cells

For the development of endothelial monolayers, polyethylene terephthalate transwell inserts were used (23.1 mm transparent membrane, pore size 0.4 μm, Corning, New York, USA), coated with 2% gelatin (w/v) in sterile PBS. For the coating, 1 ml sterile gelatin solution was added at the apical side of the insert and incubated at 37 ˚C for 30 minutes. Subsequently, remaining gelatin was removed and membranes were air dried in sterile conditions for 1.5 hours with the well plate lid slightly open. Coated inserts were stored at 4 ˚C until use but not for longer than 2 weeks. Prior to cell seeding coated inserts were thermally equilibrated with culture medium at room temperature for 15 minutes and cells were added at density of 0.12 x 10^6 cells/cm^2. Medium was added at apical/basolateral volumes of 1.5/2.7 ml. Cells were cultured for 4 days prior to the experiment and medium was refreshed the first and third day after seeding.

SI-1.3. USMB treatment protocol of endothelial cells

In the experiments with endothelial cells HBSS was replaced by EBM-2 medium. As observed in preliminary experiments, HBSS disrupts the integrity of the endothelial monolayer.

SI-1.4. Determination of barrier integrity and cell viability after USMB treatment

To determine the effect of USMB on the integrity of endothelial and epithelial barriers, microscopy images of cell monolayers were acquired after USMB treatment. Since endothelial cells were cultured on transparent transwell membranes, brightfield microscopy images were acquired before and after exposure to USMB (P_{neg} 0-0.7 MPa). On the other hand, epithelial cells cultured on translucent transwell membranes were fixed with 4% PFA in PBS (w/v) for 10 minutes at room temperature after exposure to USMB (P_{neg} 0.5-0.7 MPa), stained with DAPI and imaged with a fluorescent microscope (Leica TCS SP8 X, Leica, Amsterdam, the Netherlands) at 10x and 63x magnification (excitation 360 nm; emission 410-480 nm).

The effect of USMB treatment on the viability of epithelial cells was examined using the AlamarBlue assay. Cells were exposed to USMB at pressures 0-0.7 MPa. Immediately after USMB treatment, cells at the apical side were exposed to 0.5 ml of 500 μM resazurin sodium salt (Sigma-Aldrich) solution in PBS. Cells were incubated for 120 minutes at 37 °C at low-speed shaking at 170 rounds per minute (Heidolph Titramax 1000 incubator and shaker, Heidolph, Lelystad, the Netherlands). Next, the solution from the apical side was removed and fluorescence intensity was analyzed with a spectrofluorophotometer (FP 8300, Jasco) at excitation and emission wavelengths of 571 nm and 584/5 nm (center wavelength/bandwidth), respectively. Cell viability of each group was calculated as percentage fluorescent signal relative to that of sham treated cells.

SI-1.5. Permeability experiments with endothelial cells and model drugs

The protocol followed for the permeability experiments using endothelial cells was the same as with epithelial cells (Section 2.4), but culture medium was used instead of HBSS. In preliminary experiments it was observed that HBSS induced detachment of endothelial cells.

SI-2. Results

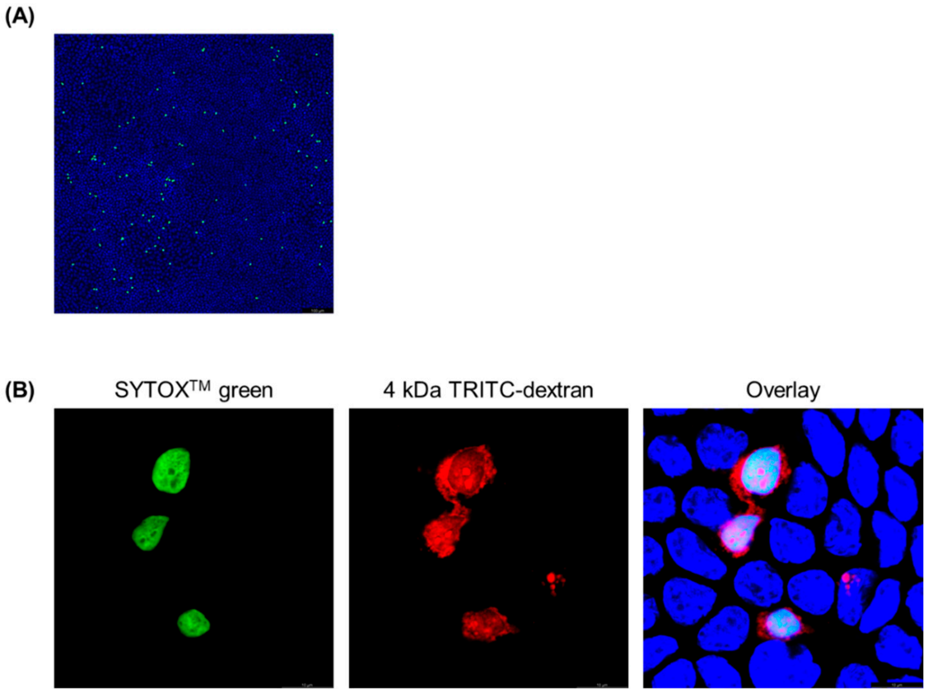

Figure S1. (A) Fluorescence image of epithelial (MDCK II) cells showing an area with intracellular accumulation of SYTOX™ green after treatment with USMB at 0.7 MPa. Cell nuclei stained with DAPI. Scale bar: 100 µm **(B)** SYTOX™ green positive cells (left) with simultaneous uptake of TRITC-dextran (middle) and overlay with DAPI (right). Scale bar: 10 µm.

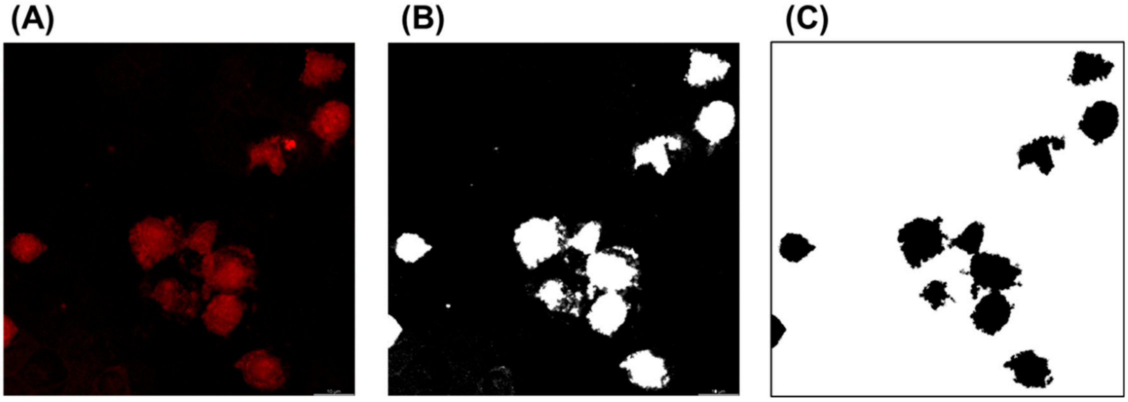

Figure S2. Summary of steps followed for the quantification of intracellular accumulation of TRITC-dextrans in epithelial (MDCK II) cells. **(A)** Raw RGB image used to calculate fluorescence intensity in the regions of interest, **(B)** Thresholding of RGB image, **(C)** generation of mask with regions of interest (black areas). Scale bar: 10 µm.

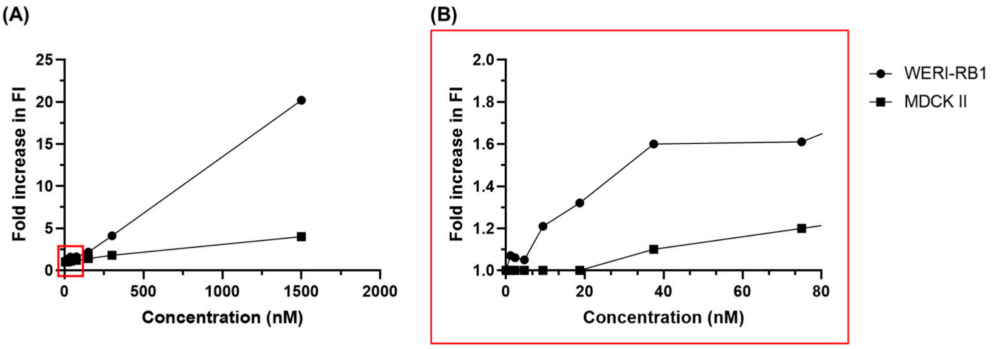

Figure S3. (A) Binding of anti-CXCR4 nanobody to retinoblastoma (WER-RB1) and epithelial (MDCK II) cells for nanobody concentrations of 0-1500 nM. **(B)** Magnification of area shown in red box in (A). FI: fluorescence intensity.

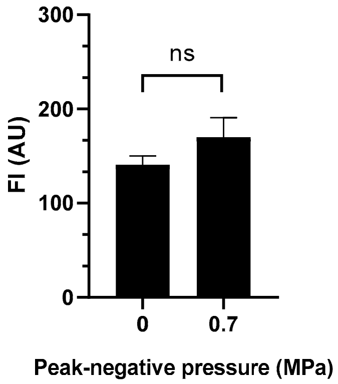

Figure S4. Binding of permeated anti-CXCR4 nanobody in retinoblastoma cells measured with flow cytometry. Nanobody was collected from the basolateral side of epithelial barriers treated with USMB at 0 or 0.7 MPa. ns: not significant ($n = 5$).

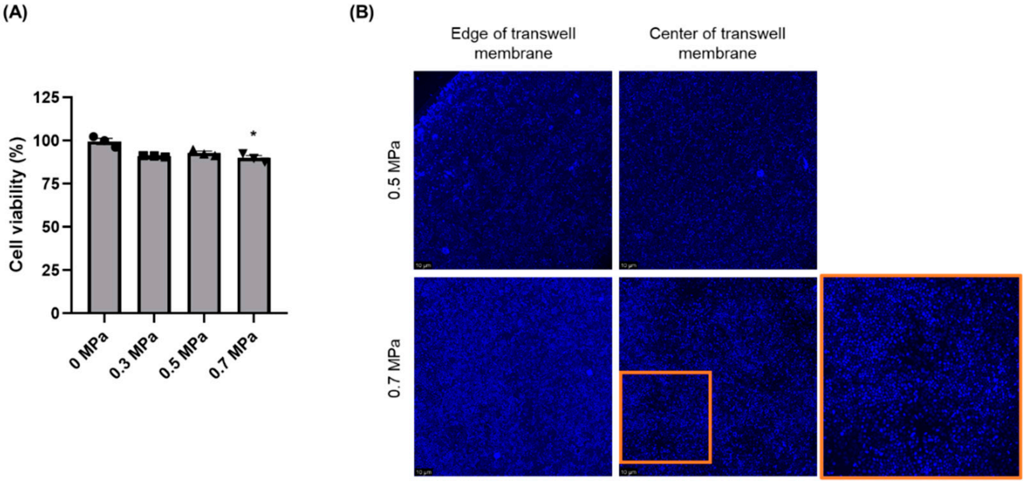

Figure S5. (A) Percentage of viable epithelial cells exposed to USMB at different ultrasound pressures (0.3-0.7 MPa) as compared to sham treated cells (n=3). Cell viability was studied using the alamarBlue™ (ThermoFisher Scientific) assay after incubation of cells with resazurin solution for 120 minutes. **(B)** Fluorescence microscopy images of epithelial cell nuclear staining (DAPI) acquired at the edge (left column)

and center (right column) of transwell membranes. No alterations in the integrity of the epithelial barrier were seen at 0.5 or 0.7 MPa. Uniform cell distribution without cell detachment was observed.

SI-2.1. The effect of USMB on endothelial barrier integrity and permeability of different molecules

USMB treatment of endothelial cells at acoustic pressures equal to and higher than 0.6 MPa disrupted the endothelial barriers (figure 7, A), and therefore these pressures were excluded from the permeability study.

Unlike the epithelial barrier, USMB did not have any significant effect on the permeability of molecules across the endothelial barrier at any of the ultrasound pressures examined. Overall, comparing the permeability of the endothelial and epithelial barrier at sham treatment (figure 2 vs. supplementary figure 7B, blue bars) revealed that the P_{app} was 22 times higher for the 6-carboxyfluorescein (1.33 x 10^{-6} cm/s for endothelial barrier; 0.06 x 10^{-6} cm/s for epithelial barrier), 26 times for the 4 kDa dextran (6.26 x 10^{-7} cm/s for endothelial barrier, 0.24 x 10^{-7} cm/s for the epithelial barrier), and 20 times for the 20 kDa dextran (2.31 x 10^{-7} cm/s for endothelial barrier, 0.12 x 10^{-7} cm/s for the epithelial barrier). This indicates that endothelial cells form a leakier monolayer than epithelial cells. This is confirmed by the percentage of permeated amount of molecules across the endothelial monolayer. Specifically, 120 minutes after compound injection for the control samples, the average permeated amount of 6-carboxyfluorescein across the endothelial monolayer was almost the same as the epithelial barrier (4.06% vs. 5.14%, figure 2 vs. supplementary figure 7B, orange bars). This difference is 8 times for the 4 kDa dextran (2.05 % for endothelial barrier, 0.26% for epithelial barrier) and 10 times for the 20 kDa dextran (0.79% for endothelial barrier, 0.08% for epithelial barrier).

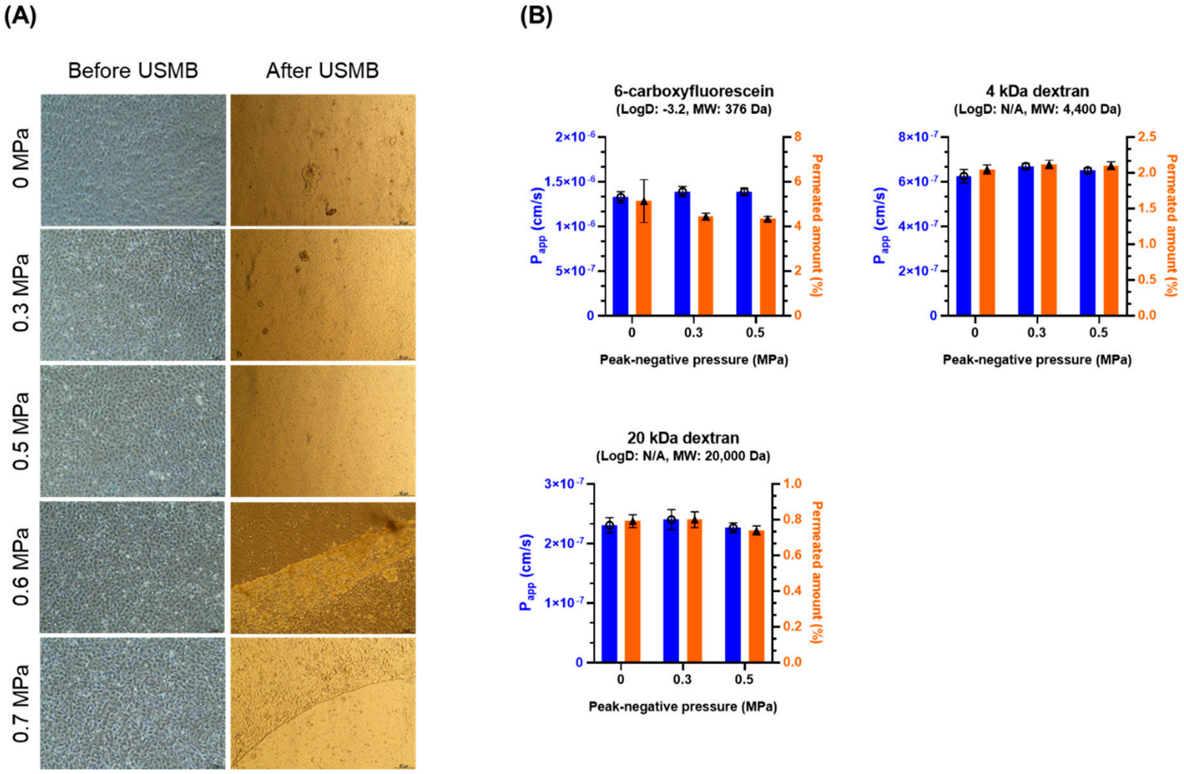

Figure S6. (A) Brightfield microscopy images of endothelial (HUVEC) barriers acquired before (left) after (right) exposure to USMB. Treatment of cells at acoustic pressures of 0.6 and 0.7 MPa resulted in extensive cell detachment and disruption of monolayer integrity. **(B)** Apparent permeability coefficient (blue bars, ○) and total amount permeated 120 minutes post-treatment (orange bars, ▲) of 6-carboxyfluorescein, 4 kDa dextran and 20 kDa dextran after treatment of endothelial cell monolayer with USMB at various acoustic pressures (*n* = 3).
